# Andrographolide protects bone marrow mesenchymal stem cells against glucose and serum deprivation under hypoxia via the NRF2 signaling pathway

**DOI:** 10.1186/s13287-022-03016-6

**Published:** 2022-07-18

**Authors:** Yanting Sun, Hao Xu, Bin Tan, Qin Yi, Huiwen Liu, Tangtian Chen, Han Xiang, Rui Wang, Qiumin Xie, Jie Tian, Jing Zhu

**Affiliations:** 1 Department of Pediatric Research Institute, Children’s Hospital of Chongqing Medical University, National Clinical Research Center for Child Health and Disorders, Ministry of Education Key Laboratory of Child Development and Disorders, Chongqing Key Laboratory of Pediatrics, Chongqing, 400014 China; 2 Department of Clinical Laboratory, Children’s Hospital of Chongqing Medical University, Chongqing, China; 3 Department of Cardiovascular (Internal Medicine), Children’s Hospital of Chongqing Medical University, Chongqing, China

**Keywords:** BMSCs, NRF2, Andrographolide, Apoptosis, Oxidative stress

## Abstract

**Background:**

Bone marrow mesenchymal stem cell (BMSCs) therapy is an important cell transplantation strategy in the regenerative medicine field. However, a severely ischemic microenvironment, such as nutrient depletion and hypoxia, causes a lower survival rate of transplanted BMSCs, limiting the application of BMSCs. Therefore, improving BMSCs viability in adverse microenvironments is an important means to improve the effectiveness of BMSCs therapy.

**Objective:**

To illustrate the protective effect of andrographolide (AG) against glucose and serum deprivation under hypoxia (1% O_2_) (GSDH)-induced cell injury in BMSCs and investigate the possible underlying mechanisms.

**Methods:**

An in vitro primary rat BMSCs cell injury model was established by GSDH, and cellular viability, proliferation and apoptosis were observed after AG treatment under GSDH. Reactive oxygen species levels and oxidative stress-related genes and proteins were measured by flow cytometry, RT-qPCR and Western blotting. Mitochondrial morphology, function and number were further assessed by laser confocal microscopy and flow cytometry.

**Results:**

AG protected BMSCs against GSDH-induced cell injury, as indicated by increases in cell viability and proliferation and mitochondrial number and decreases in apoptosis and oxidative stress. The metabolic status of BMSCs was changed from glycolysis to oxidative phosphorylation to increase the ATP supply. We further observed that the NRF2 pathway was activated by AG, and treatment of BMSCs with a specific NRF2 inhibitor (ML385) blocked the protective effect of AG.

**Conclusion:**

Our results suggest that AG is a promising agent to improve the therapeutic effect of BMSCs.

**Supplementary Information:**

The online version contains supplementary material available at 10.1186/s13287-022-03016-6.

## Introduction

As an important source for cell therapy, mesenchymal stem cells (MSCs) have been widely investigated on account of their low immunogenicity and multidirectional differentiation capability [1]. Bone marrow mesenchymal stem cells (BMSCs) are derived from bone marrow and have been widely studied for their convenience. BMSCs can secrete a variety of soluble cell factors [2] that are important to regenerative medicine, such as for immune regulation, tissue repair and angiogenesis, and are widely accepted for experimental research or clinical trials in various ischemic diseases [3–5]. Although it has been widely reported that BMSCs have homing characteristics and can selectively target the site of injury to exert therapeutic effects [6], reduced blood flow (nutrient depletion) at the site of ischemic injury and an anoxic microenvironment cause oxidative stress in BMSCs after transplantation [7–9], and a high level of reactive oxygen species (ROS) is not conducive to the survival of BMSCs [10]. Cell death is mainly mediated by mitochondrial function, and it has been reported that ischemic conditions can induce changes in the mitochondrial morphology and thus lead to the apoptosis of transplanted BMSCs [11]; moreover, the mitochondrial membrane potential is significantly reduced [12]. As reported, the number of mitochondria was obviously reduced when they were damaged [13]. Thus, effective treatments remain elusive, and improving the survival of BMSCs under glucose and serum deprivation under hypoxia (GSDH) is crucial to improving the effectiveness of BMSCs.

Currently, methods for improving the efficacy of MSC therapy include gene modification, such as overexpressing AKT (a serine/threonine kinase) [14] and insulin-like growth factor-1 (IGF-1) [15, 16], to improve the angiogenic and antiapoptotic ability of BMSCs and cell preconditioning, such as hypoxic preconditioning [17, 18], combined with small molecule compounds given before cell transplantation to improve the inflammatory microenvironment [19]. In our study, we focused on utilizing small molecule compounds to improve the effectiveness of BMSCs under GSDH. Andrographolide (AG), a natural diterpene lactone compound, has marked anti-inflammatory [20] and antitumor activity [21]. Recently, the effect of AG in ischemic disorders was extensively investigated [22, 23]. It has been reported that the function of AG is closely related to its structure. AG reacts with the Cys62 residue of the nuclear factor kappa-light-chain-enhancer of activated B cells (NF-κB) p50 subunit and plays an anti-inflammatory role [24]. In addition, it has been widely reported that AG resists oxidative injury via the nuclear factor erythroid 2-related factor 2 (NRF2) pathway in many disease models [25–28]. The NRF2 pathway is a critical signaling pathway for preventing inflammation and oxidative damage. Under oxidative damage conditions, NRF2 translocases into the nucleus, binds with antioxidant response elements (AREs), and then activates downstream antioxidant genes to regulate cellular oxidative stress and inflammation [29]. A study confirmed that AG protects neurons against oxidative damage by activating NRF2/HO-1, which reduces cellular ROS production [30]. Mitochondria are the main organelles that produce ROS, and the mitochondrial apoptosis pathway is significantly activated under serum and glucose deprivation [31]; therefore, protecting mitochondria may be a good strategy to improve the survival and retention of BMSCs. Reports have shown that AG may protect neurons against cell damage by inhibiting mitochondrial fission [32] and ameliorate Alzheimer’s disease (AD)-related gene expression by inhibiting mitochondrial swelling [33]. However, it remains unclear whether AG has a protective effect on primary rat BMSCs transplanted in ischemic-hypoxic diseases. Therefore, we utilized GSDH treatment to simulate the ischemic-hypoxic disease microenvironment. In this study, we hypothesized that AG can affect the survival of primary rat BMSCs under GSDH in vitro. Here, we report that AG protects the function of BMSCs under GSDH by reducing apoptosis, oxidative stress and mitochondrial damage. Our results also suggested that the NRF2 signaling pathway is indispensable in this process. The purpose of our study was to provide an experimental basis for improving the therapeutic effect of BMSCs.

## Materials and methods

### Materials

Sprague–Dawley rats (50–60 g) were housed at Chongqing Medical University. DMEM/F12 (4.5 g/L glucose), DMEM (1.0 g/L glucose), fetal bovine serum (FBS), and 0.25%-EDTA trypsin were all obtained from Gibco (NY, USA). AG (purity > 99%, Ruifensi, China) was dissolved in DMSO (final concentration ≤ 0.1%).

### Isolation and treatment of BMSCs

Primary rat BMSCs were isolated as reported [34], collected in DMEM/F12 containing 10% FBS and cultured at 37 °C and 5% CO2. BMSCs were used at passages 3 to 7 in this research. The BMSCs were pretreated ﻿with AG at various concentrations (2, 4, or 6 μM) for 24 h and then treated with GSDH for 24 h.

### Immunophenotype analysis of BMSCs

The BMSCs cell precipitate was collected. After centrifugation, the cells were incubated with CD34-FITC, CD45-FITC, CD11b-FITC, CD44-FITC, CD73-FITC, CD90-FITC, CD105-FITC and IgG-FITC primary antibodies (all from BD Biosciences; USA) for 15 min. Immunophenotype analysis of BMSCs was performed by flow cytometry (BD FACS Canto) [35].

### Trypan blue exclusion assay

Cell precipitates were stained with 0.1% trypan blue (Beyotime, Shanghai, China) as described. The live cell rate (%) was calculated as the number of living cells (trypan blue-negative cells)/the total cell number [36].

### Crystal violet staining

BMSCs were treated as described previously, fixed and stained with 1% crystal violet dye (Beyotime, China) for 20 min, and pictures were taken under a microscope [37]. After that, the cells were decolorized with an equal volume of 75% alcohol, and the absorbance was read at 570 nm.

### Cell counting kit‑8 assay

BMSCs were pretreated as described previously. Subsequently, the supernatant was discarded, and 10% CCK‑8 solution (Dojindo, Japan) was added [38]. After 3 h, the absorbance was measured at 450 nm.

### Hoechst 33,342 staining

After treatment, cells were stained with Hoechst 33,342 (Beyotime, China) for 10 min, and morphological changes in the nuclei were observed under a fluorescence microscope [39].

### Flow cytometric evaluation of the cell cycle

Cell precipitates in different groups were collected in a 5-ml tube. Then, the cell precipitates were treated with 75% ethanol for 24 h (4 °C), centrifuged for 5 min, incubated with RNase and propidium iodide solution (BD, USA) for 30 min (37 °C), and evaluated by flow cytometry (BD FACS Canto) [40].

### Flow cytometric evaluation of apoptosis

Cells were collected as previously described. Annexin V-FITC/PI and Annexin-PE/7-AAD kits (BD, USA) were used to detect cell apoptosis [41]. And cell apoptosis was evaluated by flow cytometry (BD FACS Canto).

### ROS production assay

BMSCs were treated with AG as previously described, incubated with DCFH-DA (10 µM, Beyotime, China) for 1 h (37 °C), and then washed three times. The fluorometric intensity was observed by fluorescence microscopy (EVOS, M7000, Invitrogen) and flow cytometry (BD FACS Canto) [42].

### Adenosine triphosphate (ATP) production assay

Cellular ATP levels were measured with an Enhanced ATP Assay Kit (Beyotime, China) [43]. BMSCs were lysed with ATP lysis buffer and centrifuged for 5 min at 4 °C and 12,000× g. The ATP content was then assayed according to the instructions of the kit. The production of ATP was analyzed with a luminometer, and the data were standardized to protein concentrations.

### Assessment of the mitochondrial content and distribution

MitoTracker Green (200 nM, Beyotime, China) solution was incubated with BMSCs for 40 min. The content of mitochondria was determined by flow cytometry (BD FACS Canto), and the distribution of mitochondria was observed by a laser confocal microscope [44].

### RT-qPCR

After treatment, total RNA was extracted by using an RNA rapid extraction kit (BioFlux, China). cDNA was synthetized with a PrimeScript RT reagent kit (TaKaRa Biotechnology, Japan), and the procedure was performed as reported [45]. Gene expression was quantified by using TB Green (TaKaRa Biotechnology, Japan). The primer sequences used are given in Table 1.

**Table 1:** Primer sequences

Name	Primer sequence (5′–3′)
HK1	F: CAGACGAACCTGGACTGTGGAATC R: TCCTCTTCACCGCATCCCTCAG
PKM	F: GTGCCGCCTGGACATTGACTC R: ATTCAGCCGAGCCACATTCATCC
LDH1	F: GCTCATCGTCTCAAACCCAGTGG R: ACTCCCAGCCTTTCTCCCATCAG
G6PD	F: CGCTCACGACTCACAGTGGATG R: AGGTGCTTGTAGGAGGCTGGATC
TKT	F: GCACCAACCAACAGCCATCATTG R: CATGCCACGCCTCCTTGTCTTC
THLDO1	F: CGCTGGCAGGCTGTGATTTCC R: GTCACTGGTCTGGGCTGCTTTG
CS	F: GAACTCATCCTGCCTCGTCCTTG R: CTGTCTTCCCATGCTGCTGTCTG
IDH1	F: ATGGCGGTTCTGTGGTGGAAATG R: GGTCATTGGTGGCATCCCGATTC
OGDH	F: CCGTGCCCGCTGACATTATCTC R: CCGATGAAAGTGGTGGTGGGTAAG
HO-1	F: CAGACAGAGTTTCTTCGCCAGAGG R: TGTGAGGACCCATCGCAGGAG
NQO-1	F: AGGCTGCTGTGGAGGCTCTG R: GCTCCCCTGTGATGTCGTTTCTG
GSH-px	F: TGCAATCAGTTCGGACATCAGGAG R: CTCACCATTCACCTCGCACTTCTC
CAT	F: AGCGGATTCCTGAGAGAGTGGTAC R: CTGTGGAGAATCGGACGGCAATAG
GCLC	F: GCACATCTACCACGCAGTCAAGG R: AGAACATCGCCGCCATTCAGTAAC
GAPDH	F: ATGGCTACAGCAACAGGGT R: TTATGGGGTCTGGGATGG
ND1	F: CGAGCCGTTGCCCAAACCATC R: AGGGAGAAGGAGCCGCTTATTAGG
ACTB	F: AGATCAAGATCATTGCTCCTCCT R: ACGCAGCTCAGTAACAGTCC

### Mitochondrial DNA (mt-DNA) copy number

Genomic DNA was extracted with a gDNA Assay Kit (Bio Flux, China) and analyzed with TB Green (TaKaRa Biotechnology, Japan) [46]. The mitochondrial DNA (mt-ND1) to nuclear DNA (ACTB) ratio was calculated to determine the mitochondrial DNA copy number.

### Lactic acid level and HK activity determination

The production of lactic acid was detected by using a lactic acid assay kit and HK assay kit (Solarbio, China). The cells were lysed in an ice bath by ultrasonication (300 W, 3 min) and centrifuged for 10 min (12,000× g, 4 °C). The extraction solution was added to the supernatant and centrifuged again according to the previous conditions. The absorbance was measured at 450 nm and 340 nm after the supernatant was added to the test solution according to the instructions. The lactic acid and HK content data were normalized to the protein concentrations [47].

### Western blotting analysis

Cell lysates were extracted with a Whole/Nucleus/Cytoplasm Protein Extraction Kit (Beyotime, China) according to the manufacturer’s protocol. Proteins were separated on 10% SDS‑PAGE gels (Gibco, USA) and transferred to polyvinylidene fluoride membranes (Millipore, USA) as reported [48]. After blocking for 1 h, the cells were incubated overnight at 4 °C with primary antibodies against STAT3, p-STAT3, p-AKT, t-AKT, p-FOXO1, t-FOXO1, 14-3-3ζ/δ, p-S6 (1:1000, Cell Signaling Technology, USA), NRF2 (1:2000, Abcam, USA), HO-1, NQO-1, histone H3 (1:1,000, Proteintech Group, China), GCLC, GPX4, CAT, SOD1 (1:800, Hua Bio, China), and β-actin (1:1000, Zhongshan Company, China). Subsequently, the membranes were incubated with a goat anti-mouse/rabbit (1:5000, ZSGB-BIO, China) antibody for 1 h at room temperature and visualized under the ChemiDoc Touch Imaging System (Bio-Rad Laboratories Inc., USA). The results were analyzed by ImageJ software.

### Statistical analysis

The data are expressed as the mean ± standard deviation, and all experiments were performed at least three independent times. For data conforming to a normal distribution, one-way ANOVA was used to compare differences between multiple groups, and t-tests were used to detect differences between two groups. Statistical analyses were performed using GraphPad Prism 8.0 software (GraphPad Software Inc., USA). A *P* value of < 0.05 was considered statistically significant.

## Results

### Characterization of BMSCs and construction of the cell model

**Fig. 1:**
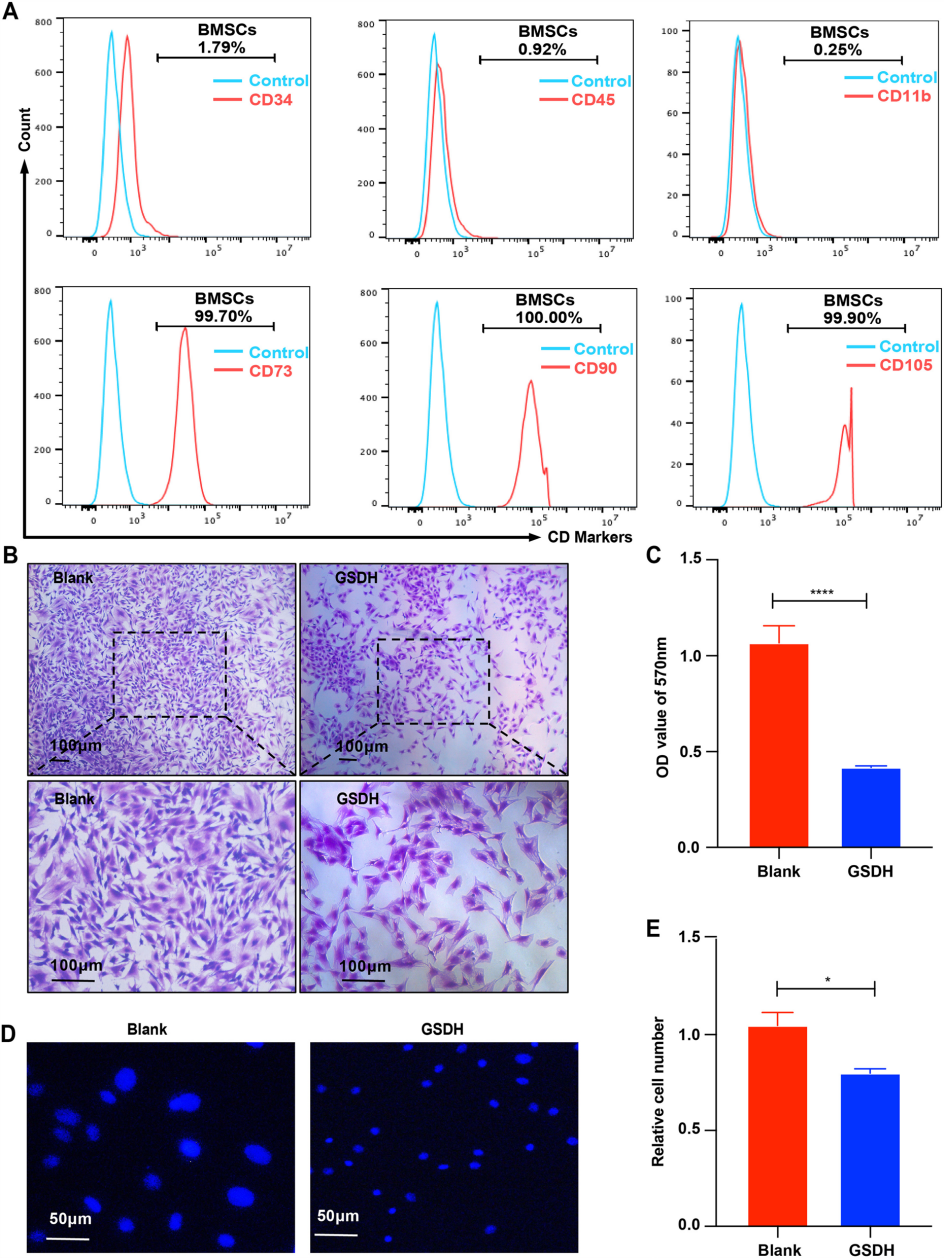
Characterization of BMSCs and validation of the cell injury model. **A** Flow cytometric analysis of CD markers. **B**, **C** Crystal violet staining images and the OD value at 570 nm of the Blank group and GSDH group (GSDH treated for 24 h), scale bars: 100 μm. **D** Hoechst 33,342 staining. Scale bars: 50 μm. **E** Number of live cells detected by trypan blue staining (*n* = 3), scale bars: 50 μm. *P < 0.05, ****P < 0. 0001

The steps used to extract primary rat BMSCs are shown in Additional file 1: Fig. S1A. The cell morphology was observed under a microscope. At passage 1, we found many red blood cells, fat droplets and other impurities in addition to BMSCs; at passage 3, the BMSCs were spindle shaped and adherent, and the nuclei were round and located in the center of the cells (Additional file 1: Fig. S1B). The results of the flow cytometry analysis showed that over 99% of cells expressed CD73, CD90, or CD105 and that fewer than 2% of the cells expressed CD34, CD45, or CD11b (Fig. 1A). These results demonstrated that BMSCs were successfully isolated. Then, we constructed a cell damage model by exposing BMSCs to GSDH. Crystal violet staining showed that the density of BMSCs and the OD value at 570 nm were significantly decreased after GSDH induction (Fig. 1B, C). Furthermore, most of the nuclei exhibited nuclear pyknosis (Fig. 1D), and the number of living cells was decreased in the GSDH group. Thus, we successfully ﻿characterized primary rat BMSCs and constructed a cell damage model.

### Proliferation protective effects of AG in BMSCs under GSDH

**Fig. 2:**
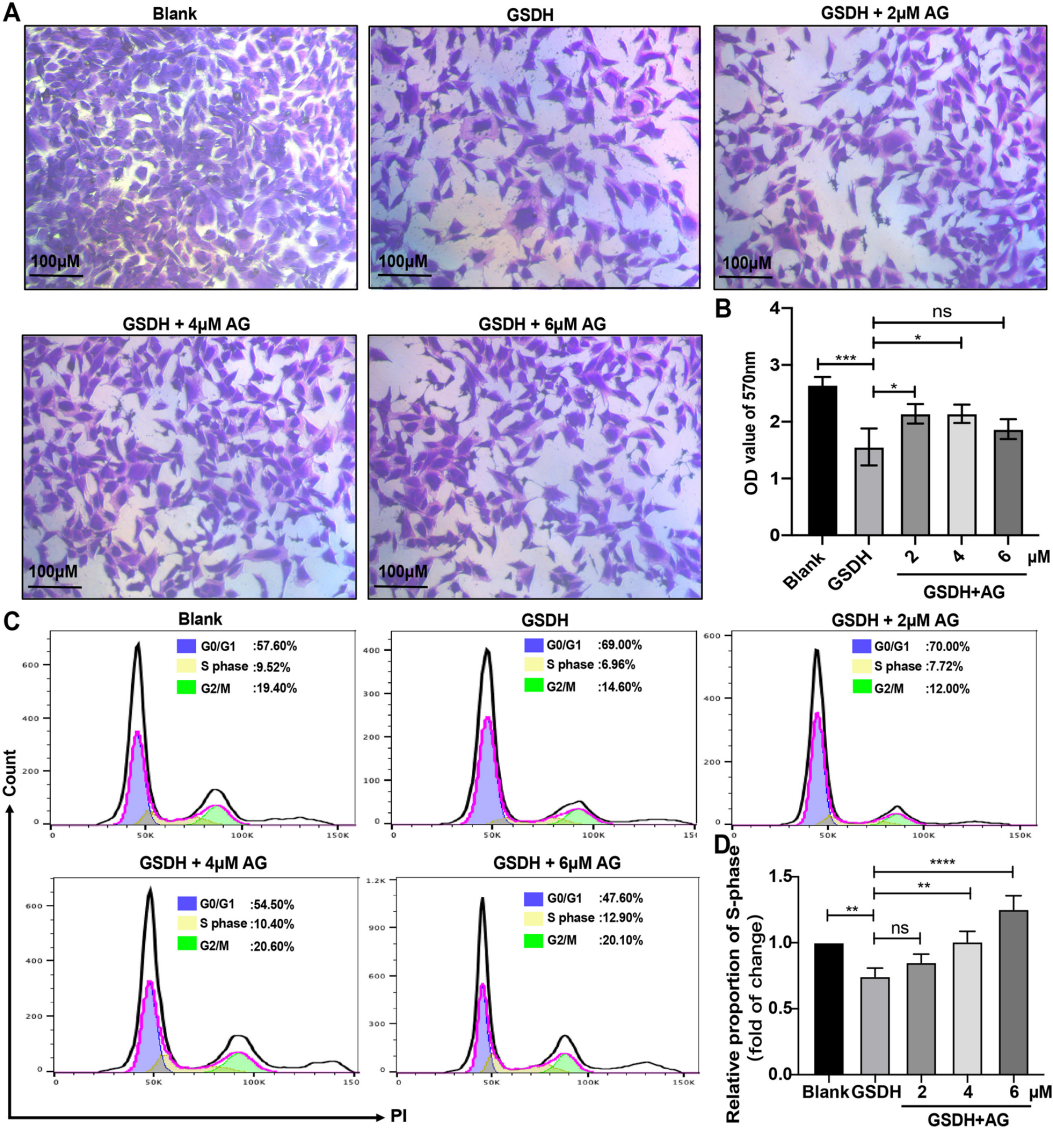
The effects of AG on proliferation in BMSCs under GSDH. **A** Crystal violet staining and **B** measurement of the OD value at 570 nm were used to assess the proliferation of BMSCs (*n* = 5). **C** PI staining was used to measure the cell cycle. **D** Quantitative analysis of the DNA content distribution using flow cytometry (*n* = 3). *P < 0.05, **P < 0. 01, ***P < 0. 001, ****P < 0.0001

To assess the protective effect of AG (the chemical structural formula of which is shown in Additional file 2: Fig. S2A), we first performed a CCK-8 assay. The data showed that cell viability was increased in the groups treated with 2 μM, 4 μM and 6 μM AG compared to the GSDH group but that there was no significant difference between the GSDH and 8 μM AG groups (Additional file 2: Fig. S2B); therefore, we used AG at concentrations of 2 μM, 4 μM and 6 μM in subsequent experiments. Cell proliferation was assessed by crystal violet staining. The cell density was markedly reduced in the GSDH group, and this decrease was gradually reversed by the addition of different concentrations of AG (Fig. 2A). The quantitative results revealed that cell proliferation was increased in the groups treated with 2 ﻿μM and 4 μM AG compared to the GSDH group (Fig. 2B), and the cell cycle results revealed that AG induced S phase arrest at concentrations of 2﻿ μM, 4 μM and 6 μM (Fig. 2C, D). These results suggested that cell viability and proliferation can be significantly increased by AG under GSDH.

### AG increases the antioxidative effects on BMSCs under GSDH

**Fig. 3:**
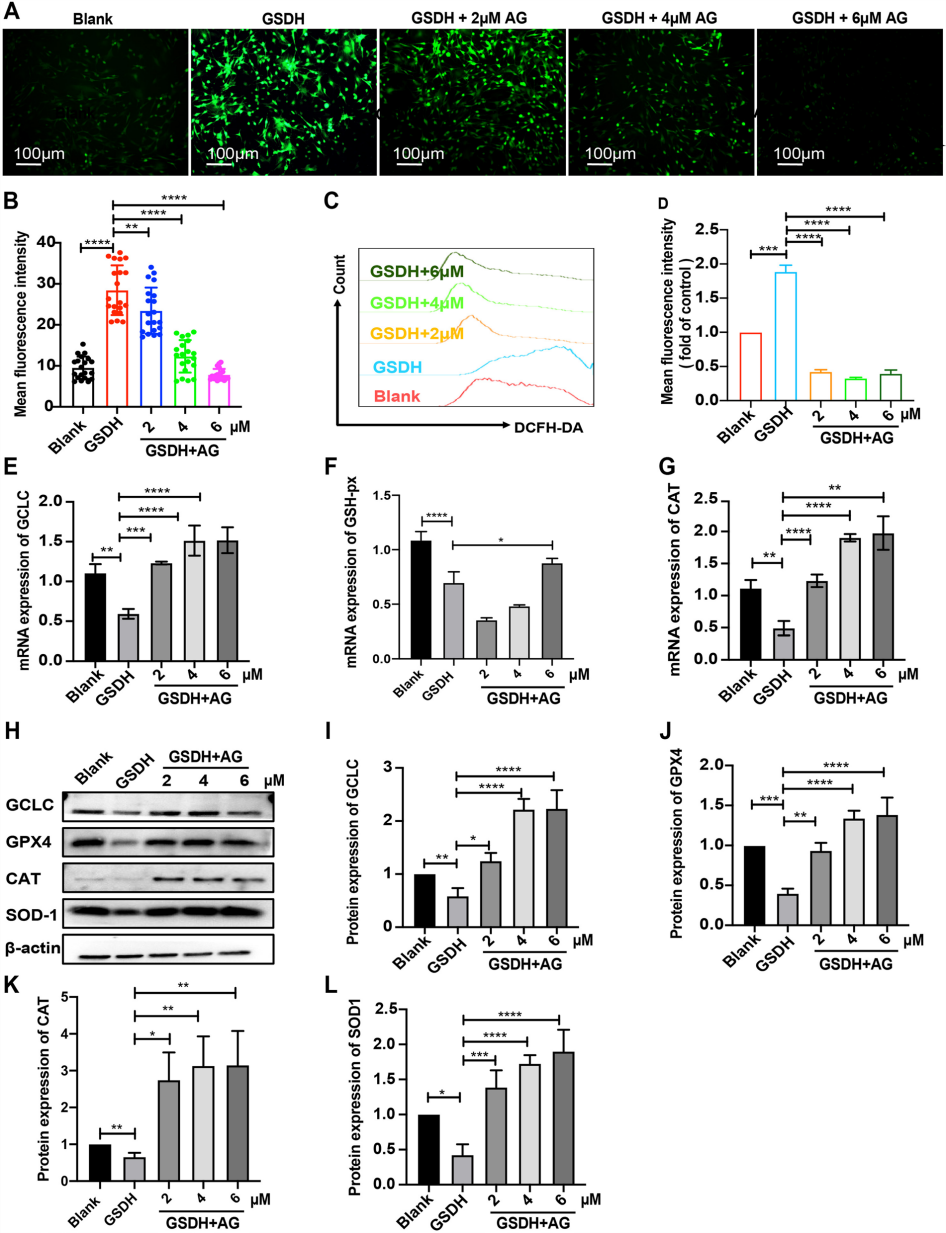
The effects of AG on antioxidative stress in BMSCs under GSDH. In (H), the samples derive from the same experiment and that the blots were processed in parallel and β-actin was used as the control. **A** DCFH-DA staining was used to measure ROS; stained cells were observed by fluorescence microscopy, and **B** statistical analysis of the MFI of ROS in each group (*n* = 20 cells). **C**, **D** Flow cytometry was used to measure the MFI of ROS in each group (*n* = 3). **E**–**G** qRT-PCR analysis of the effect of AG on antioxidation-related genes (*GCLC*, *GSH-px*, *and CAT*) in BMSCs (*n* = 3). **H** Western blot analysis of the protein levels of GCLC, GPX4, CAT and SOD1 (*n* = 3). Statistical analysis of data from each group is shown in (I-L), normalized with β-actin (*n* = 3). *P < 0.05, **P < 0.01, ***P < 0.001, ****P < 0.0001

We used DCFH-DA (a fluorescent probe for ROS) to investigate the change in intracellular ROS levels by fluorescence microscopy (Fig. 3A). Statistical analysis revealed that AG treatment alleviated GSDH-induced ROS accumulation in a dose-dependent manner (Fig. 3B). Flow cytometry was used to further examine ROS production, and our data showed that compared to GSDH, AG treatment markedly reduced the ROS content (Fig. 3C, D). Moreover, the mRNA level of antioxidative stress-related genes *(GSH-px*, *CAT* and *GCLC)* was elevated in the AG group compared to the GSDH group (Fig. 3E–G). We also indicated that GSDH treatment significantly decreased the expression of antioxidant proteins (GCLC, GPX4, CAT and SOD1), whereas AG treatment reversed these changes (Fig. 3H). The quantitative results are shown in Fig. 3I–L. Thus, these data showed that AG treatment ameliorates GSDH-induced ROS accumulation in cultured BMSCs.

### Treatment with AG attenuates GSDH-induced apoptosis and changes in the mitochondrial membrane potential (ΔΨm)

**Fig. 4:**
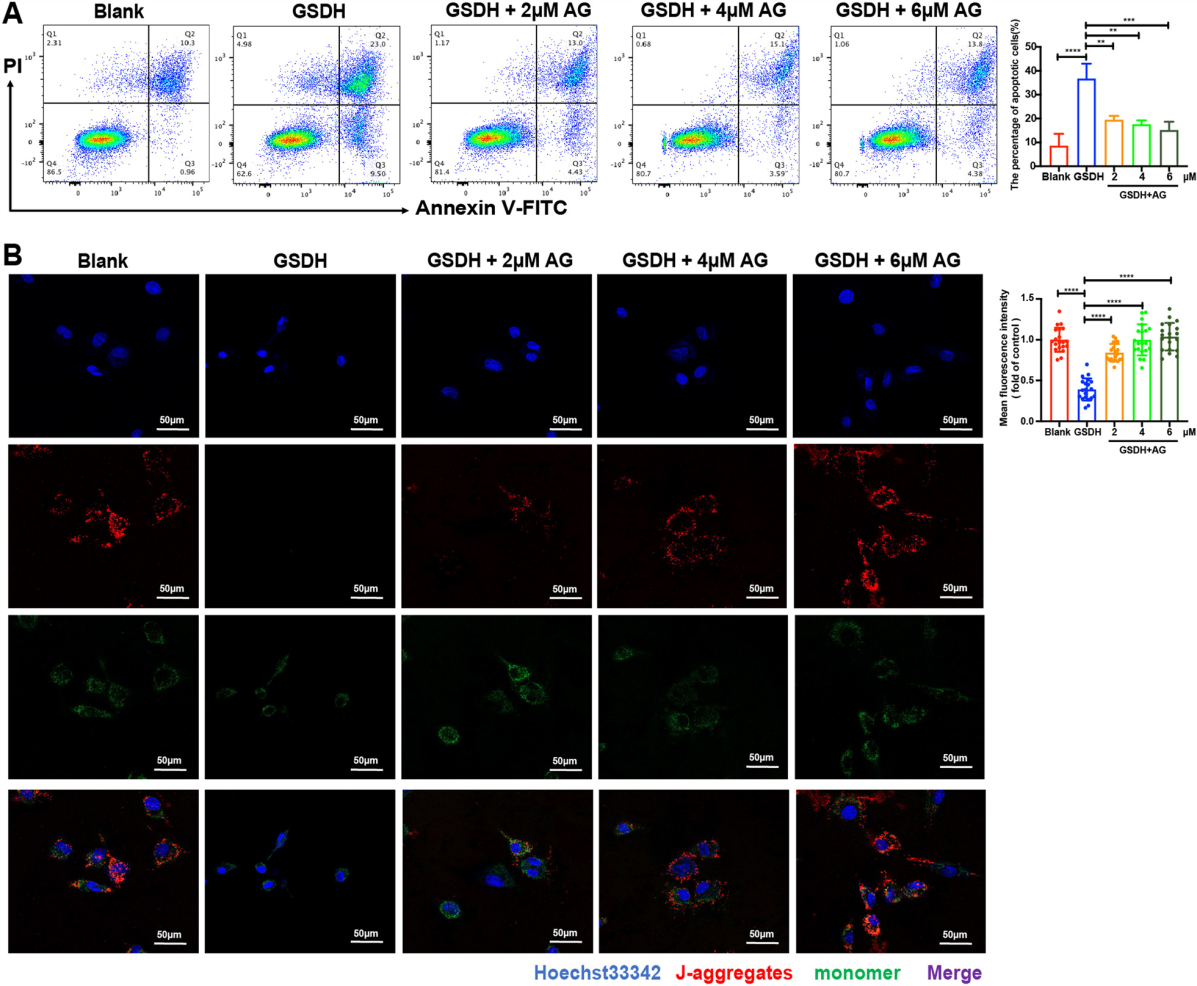
The effects of AG on GSDH-induced apoptosis and changes in ∆Ψm. **A** Flow cytometry with Annexin V-FITC and PI staining, and cells located in quadrants Q2 and Q3 were considered apoptotic cells (*n* = 3). Quantitative analysis of the apoptotic rate is shown at right. **B** The ∆Ψm was determined by JC-1 staining, Blue: Hoechst 33,342, Red: J–aggregates, Green: monomers. Statistical analysis of the red/green MFI ratio is shown at right (*n* = 20 cells). Scale bars: 50 μm. **P < 0. 01, ***P < 0. 001, ****P < 0.0001

We used flow cytometry to investigate the changes in BMSCs apoptosis. The data revealed a marked increase in the apoptosis rate in the GSDH group, which was reversed by the administration of AG (Fig. 4A). Research has found that apoptosis is often accompanied by a decline in ∆Ψm, and ∆Ψm increases when respiratory function is enhanced [49]. We further examined ∆Ψm using the fluorescence dye JC-1, which can accumulate in mitochondria. When ∆Ψm is elevated, red fluorescence is mainly produced. In contrast, predominantly green fluorescence is a sign of early apoptosis. Statistical analysis revealed that the red/green fluorescence ratio was decreased in the GSDH group, which was reversed by the administration of AG (Fig. 4B). These data suggested that AG attenuates GSDH-induced apoptosis.

### Effects of AG on the mitochondrial morphology and number

**Fig. 5:**
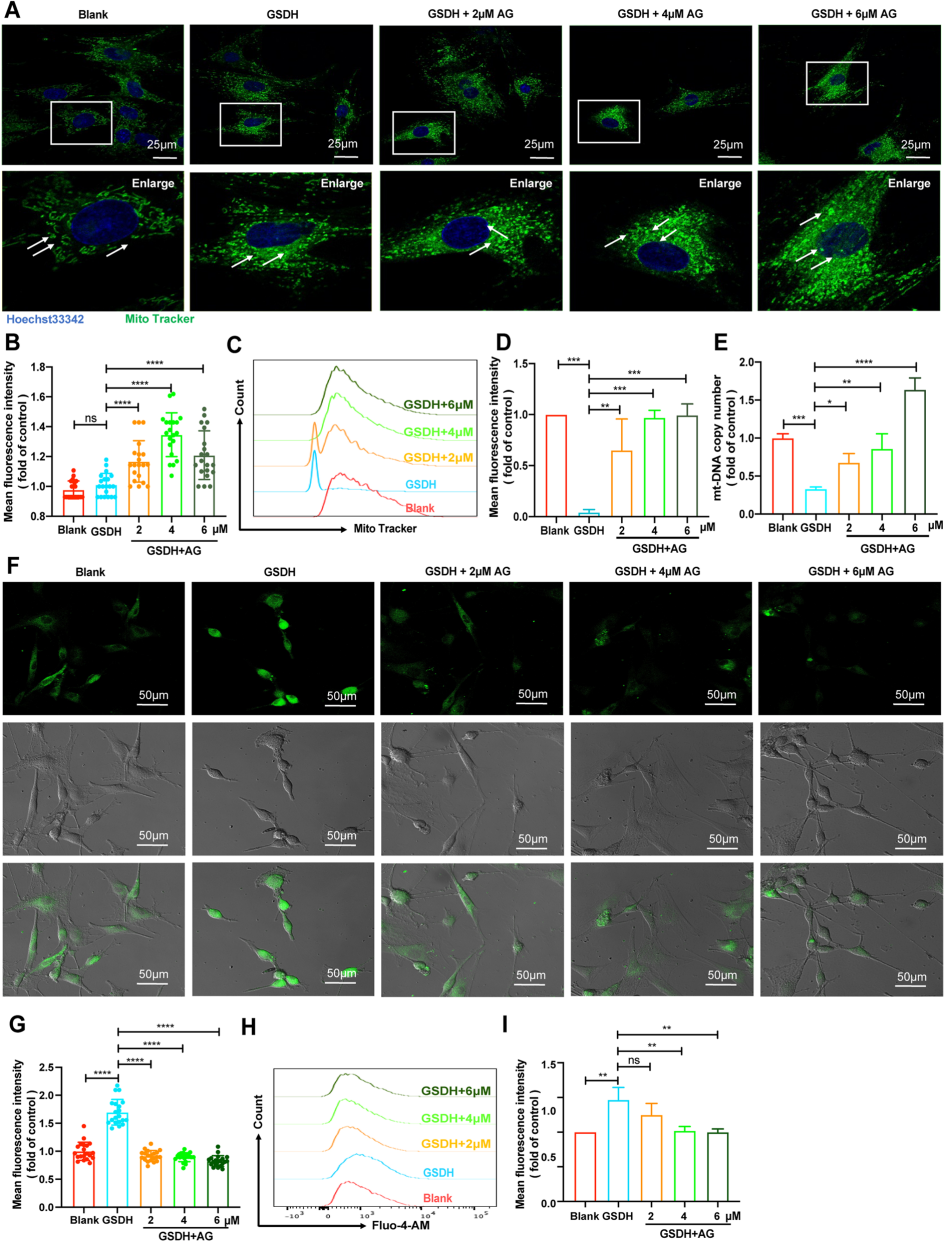
The effects of AG on the mitochondrial morphology and number. **A** The mitochondrial morphology was observed by a laser confocal microscope, Blue: Hoechst 33,342, Green: MitoTracker, scale bars: 25 μm. **B** Analysis of the MFI of each group by using ImageJ software (*n* = 20 cells). **C** The number of mitochondria was assessed by flow cytometry (*n* = 3). **D** Analysis of the MFI of mitochondria in each group. **E** qRT-PCR analysis of the mitochondrial DNA copy number. **F** Fluo-4 AM staining was used to assess Ca^2+^ by laser confocal microscopy. **G** The MFI of each group was analyzed by ImageJ software (*n* = 20 cells). **H** Cellular Ca^2+^ was determined by flow cytometry (*n* = 3). **I** Analysis of the MFI of fluo-4AM in each group. Scale bars: 50 μm. *P < 0.05, **P < 0.01, ***P < 0.001, ****P < 0.0001

It has been reported that oxidative stress impairs mitochondrial function via Ca^2+^ accumulation, leading to apoptosis and then induces toxicity in cultured dorsal root ganglion neurons [50]. As AG plays an indispensable role in inhibiting apoptosis, we next explored the change in mitochondria in BMSCs. First, the distribution and morphology of mitochondria were observed by laser confocal microscopy. We found that mitochondria were sparse in BMSCs but became dense after GSDH treatment. We also observed that AG treatment caused the mitochondria to move to around the nucleus (Fig. 5A). The mean fluorescence intensity (MFI) of MitoTracker staining is shown in Fig. 5B. Accordingly, the content of intracellular mitochondria was measured by flow cytometry (Fig. 5C), and the results also showed that the mitochondrial content was significantly increased by AG (Fig. 5D). To investigate whether AG ﻿affects the number of mitochondria in BMSCs, we evaluated the mt-DNA copy number, and the results showed that the mt-DNA copy number was significantly decreased after GSDH but increased after AG treatment (Fig. 5E). As shown in Fig. 4B, the GSDH-induced change in the ∆Ψm in BMSCs was reversed by AG. We also evaluated cellular Ca^2+^ accumulation by Fluo-4AM staining to further assess the effects of AG on mitochondrial functions and the underlying mechanism (Fig. 5F). As shown in Fig. 5G, the MFI of Ca^2+^ revealed GSDH-induced Ca^2+^ overload in BMSCs, and the addition of AG relieved the calcium overload induced by GSDH. Accordingly, the content of Ca^2+^ was measured by flow cytometry (Fig. 5H, I). These data indicated that GSDH-induced changes in the mitochondrial morphology, number and function can be reversed by AG.

### Effect of AG on the metabolic status of BMSCs

**Fig. 6:**
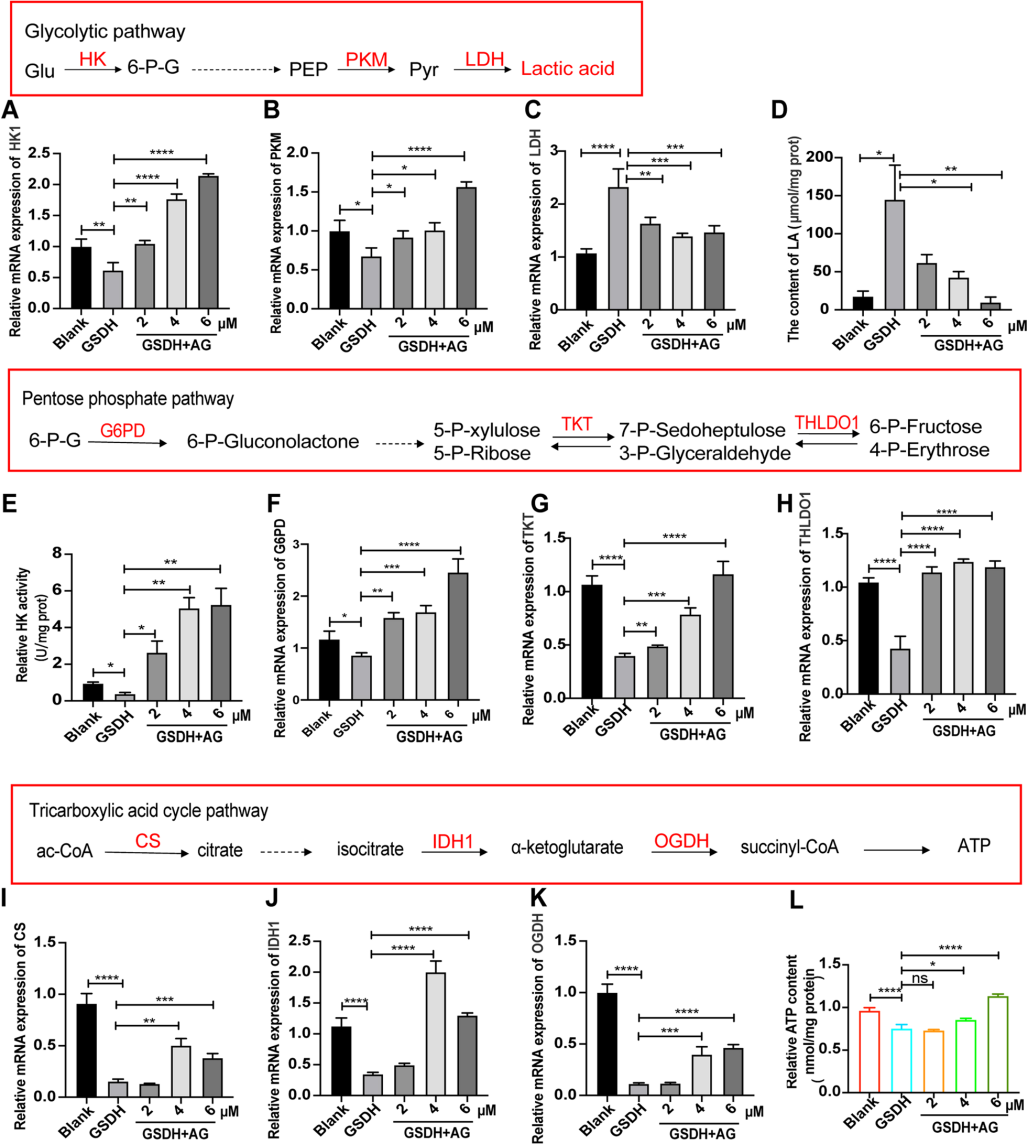
The effects of AG on the metabolic status in BMSCs under GSDH. **A**–**C** qRT-PCR analysis of the effect of AG on glycolysis-related enzymes (*HK*, *PKM* and *LDH*) in BMSCs (*n* = 3). **D** Measurement of lactate (LA) levels in BMSCs treated with AG under GSDH (*n* = 3). **E**–**H** qRT-PCR analysis of the effect of AG on pentose phosphate pathway-related enzymes (*G6PD*, *TKT* and *THLDO1*) in BMSCs (*n* = 3). **I**–**K** qRT-PCR analysis of the effect of AG on TCA-related enzymes (*CS*, *IDH1* and *OGDH*) in BMSCs (*n* = 3). **L** Measurement of ATP content in BMSCs treated with AG under GSDH (*n* = 3). *P < 0.05, **P < 0.01, ***P < 0.001, ****P < 0.0001

Mitochondria are the main sites of oxidative respiration and energy production in cells [51]. A relevant diagram of glucose metabolism is shown in Additional file 3: Fig. S3. To further determine whether the changes in mitochondrial function were related to the state of energy metabolism, we detected expression changes in key genes for glycolysis (Fig. 6A–C). The mRNA levels of *HK* and *PKM* were increased in the AG group vs. the GSDH group, the mRNA level of *LDH* was increased, and the content of lactic acid was significantly decreased (Fig. 6D). We also found that the activity of HK was increased (Fig. 6E), indicating that glucose consumption increased and glycolysis decreased. Next, we found that the expression of pentose phosphate pathway-related genes (*G6PD*, *TKT* and *THLDO1*, Fig. 6F–H) and oxidative phosphorylation pathway-related enzymes (*CS*, *IDH1* and *OGDH*, Fig. 6I–K) was significantly increased. We used the Enhanced ATP Assay Kit to assess the ATP content to further measure the energy production of BMSCs, and we found that the GSDH-induced disruption of ATP production in BMSCs was reversed by AG (Fig. 6L). These data suggested that the metabolic status of BMSCs changed from glycolysis to oxidative phosphorylation to increase the ATP supply.

### Effect of AG on the activation of NRF2 pathways in BMSCs

**Fig. 7:**
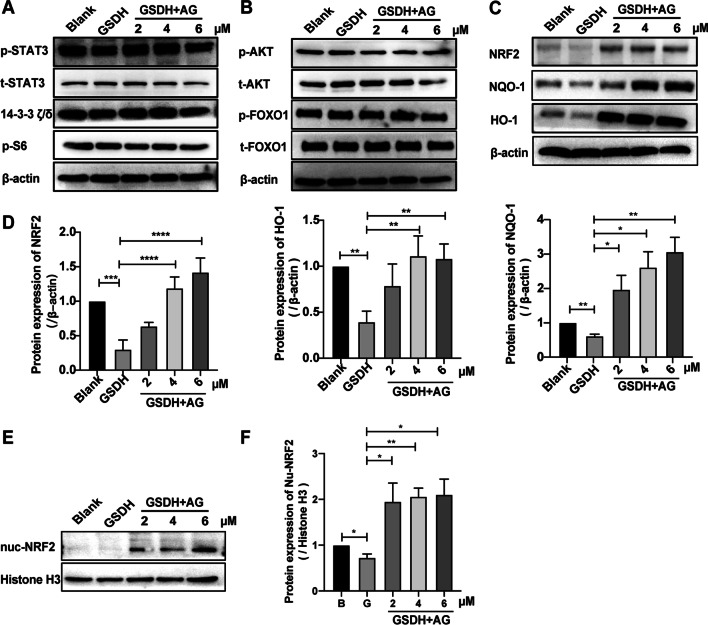
The signaling mechanism of AG in BMSCs. In **A**–**C**, **E**, the samples derive from the same experiment and that the blots were processed in parallel. In **A**–**C**, β-Actin was used as a loading control, and in **E**, Histone H3 was used as a control. **A** The expression of p-STAT3, t-STAT3, 14-3-3 ζ/δ and p-S6 was measured by Western blotting. **B** The protein levels of p-AKT, t-AKT, p-FOXO1 and t-FOXO1 were determined by Western blotting. **C** NRF2, NQO-1 and HO-1 protein expression was evaluated by Western blotting, and the statistical analysis is shown in **D**, normalized with β-actin (*n* = 3). **E** Western blot analysis of the expression of nuclear NRF2. Quantitative analysis is shown on the right, normalized with Histone H3 (*n* = 3). *P < 0.05, **P < 0.01, ***P < 0.001, ****P < 0.0001

The previously presented results confirm the efficacy of AG in inhibiting mitochondrial damage, cell apoptosis and ROS accumulation. However, the underlying mechanism remains unclear. AG is known to suppress IL-6 cell signaling, including STAT3 and AKT phosphorylation [52, 53]. We first measured the expression of proteins related to the STAT3 and AKT signaling pathways (STAT3, 14-3-3 ζ/δ, p-S6, AKT and FOXO1), but our Western blotting results showed no differences among all groups (Fig. 7A, B), and the statistical analysis is shown in Additional file 4: Fig. S4. These results indicate that these proteins are not involved in the beneficial effects of AG on BMSCs survival. Recently, it was reported that AG can protect neurons against inflammation-mediated injury by activating NRF2/HO-1 [30] and can reduce liver cell death via NRF2/HO-1 signaling [54]. Our results suggested that the levels of NRF2, NQO-1 and HO-1 were reduced after GSDH treated and that AG significantly upregulated the expression of these proteins (Fig. 7C, D). We further tested the nuclear protein expression of NRF2 and found that it was increased in the AG group vs. the GSDH group (Fig. 7E, F). The data indicated that NRF2 pathway activation was indispensable in the protective effect of AG.

### Specific inhibitors of the NRF2 pathway blocked the rescue effect of AG

**Fig. 8:**
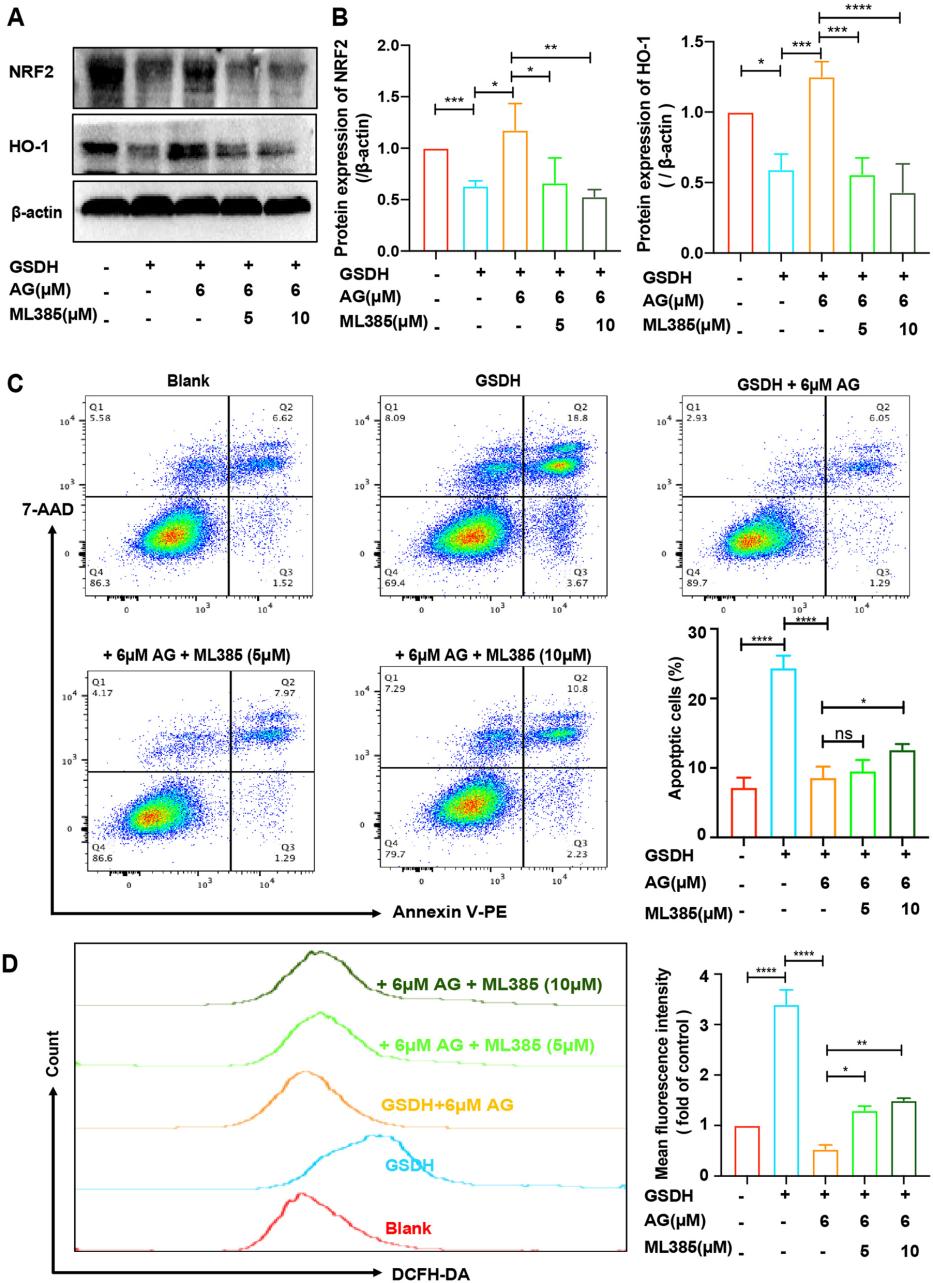
AG regulates apoptosis and oxidative stress in BMSCs under GSDH through NRF2 pathway. In **A** the samples derive from the same experiment and that the blots were processed in parallel, β-actin was used as a loading control. **A** Western blot analysis of the expression of NRF2 and HO-1, with β-actin as a control (*n* = 3), and **B** statistical analysis in each group, normalized with β-actin. **C** Flow cytometry with Annexin V-PE and 7-AAD staining (*n* = 3). Statistical analysis is shown on the right. **D** The number of DCFH-DA-positive cells was assessed by using a flow cytometer (*n* = 3), and statistical analysis of the MFI in each group is shown on the right. *P < 0.05, **P < 0.01, ***P < 0.001, ****P < 0.0001

ML385 (a specific inhibitor of NRF2) was used to investigate the mechanisms by which AG inhibits apoptosis and oxidative stress. We found that ML385 significantly inhibited NRF2 and HO-1 expression at 5 μM and 10 μM (Fig. 8A), and densitometry analysis is shown in Fig. 8B. The cell cycle results showed that S phase arrest was decreased in the ML385 group compared to the AG group (Additional file 5: Fig. S5). Additionally, ML385 significantly increased the level of ROS and the cell apoptosis rate after AG treatment (Fig. 8D, E). A schematic model of the potential mechanism by which AG improves the therapeutic effect of BMSCs under GSDH is shown in Additional file 6: Fig. S6. These findings suggested that AG protects BMSCs against apoptosis and oxidative damage via the NRF2 pathway.

## Discussion

BMSCs are one type of MSCs, and they possess the characteristics of MSCs and are ideal seed candidates for tissue repair [55]. Significant progress in myocardial injury repair [56], angiogenesis [57] and spinal cord injury repair [58, 59] has been made using BMSCs, and preclinical research on these cells has also been carried out [60]. However, differences in culture conditions, the cell course, passage methods and cell density affect the BMSCs phenotype, and the expected results were not observed in preclinical studies [61]. This may be related to impairment of cell function and an increase in cell death caused by a poor disease microenvironment [62, 63]. In our study, we used GSDH (1% O_2_) to simulate the microenvironment of BMSCs at the site of ischemic injury in vitro. Our results revealed that BMSCs showed a significant decrease in cell number and significant nuclear consolidation after 24 h of GSDH, suggesting that the microenvironment during GSDH significantly limits the survival of BMSCs (Fig. 1). Thus, we aimed to overcome this limitation by promoting the survival of BMSCs under GSDH to improve the therapeutic effect of BMSCs in our study.

AG is a natural compound with a variety of biological activities. Previous studies have shown that AG has multiple biological functions, such as anti-inflammatory [64], antitumor [65] and antioxidative stress [29] effects. Zhang X-F et al. [66] found that AG attenuates mitochondrial damage in RAW 264.7 cells. First, we performed a CCK-8 assay to confirm a safe dose range of AG in BMSCs using doses of 2, 4, 6 and 8 μM. The results showed that cell viability was significantly increased in the groups treated with 2 ﻿μM, 4 μM and 6﻿ μM AG compared to the GSDH group, but there was no significant difference between the GSDH and 8 μM AG groups (Additional file 2: Fig. S2B); therefore, we used AG at concentrations of 2 μM, 4 μM and 6 μM in subsequent experiments. Although the increase in cell numbers after 6 μM AG treatment was not statistically significant compared to the GSDH group (Fig. 2B), it was in fact increased. Additionally, the CCK-8 assay showed an increase in cell viability, and the cell cycle assay indicated an increase in the proportion of cells in S phase. Thus, we suggest that cell proliferation was increased in the groups treated with 2 μM, 4 μM and 6 μM AG compared to the GSDH group. It has been reported that ROS act as second messengers and are indispensable for cell proliferation, differentiation and survival [67] and that the function of stem cells is highly dependent on intracellular redox homeostasis [68]. In recent years, it was reported that abnormal elevation of ROS levels is detrimental to cell growth under pathological conditions due to the loss of the normal balance between ROS production and scavenging [69]. Our findings revealed that AG protected GSDH-treated BMSCs from oxidative stress induced by abnormally increased ROS levels (Fig. 3A–D). It has been reported that a high level of ROS impairs cell proliferation and induces cell death by causing cellular redox imbalance [70]. We suggest that GSDH treatment induces excessive amounts of ROS and then impairs cell proliferation. *GSH-px*, *CAT* and *GCLC* are antioxidant-related genes, and the data in Fig. 3E, G show that the mRNA expression of *GCLC* and *CAT* was increased in the groups treated with 2 μM, 4 μM and 6 μM AG compared to the GSDH group. However, although 6 μM AG treatment significantly increased the mRNA expression of *GSH-px* compared to that in the GSDH group, there was decrease rather than increase in the 2 μM and 4 μM AG groups (Fig. 3F). This result suggests that the mRNA expression of *GSH-px* was insensitive to 2 μM and 4 μM AG concentrations; the reduction in *GSH-px* mRNA expression induced by GSDH could not be ameliorated by 2 μM and 4 μM AG. However, the exact details of the explanation should be further confirmed in our future research. Furthermore, we confirmed that AG resisted oxidative damage by increasing GCLC, SOD1, CAT and GPX4 protein levels (Fig. 3H–L). These results suggested that AG has strong potential in promoting the antioxidant activity of BMSCs for application in cell therapy.

Most ROS are produced in the mitochondria, and excessive accumulation of ROS in the mitochondria induces apoptosis [71]. Therefore, we assessed the alteration in apoptosis, and the results showed that GSDH treatment, in addition to inducing oxidative stress, induced a high level of apoptosis, while AG treatment resulted in a significant decrease in the apoptosis rate (Fig. 4). Mitochondria are critical for cell survival, apoptosis and other vital activities [72], and we further found that AG alleviated the alterations in mitochondrial morphology and number in BMSCs under GSDH and increased the mitochondrial DNA copy number (Fig. 5A–E), confirming that AG treatment inhibited apoptosis in BMSCs under GSDH.

It has been shown that when large amounts of Ca^2+^ accumulate in the mitochondrial matrix, and then, Ca^2+^ is released into the cytoplasm and accumulates, triggering calcium overload and leading to cell death. The production of ROS also leads to cellular dysfunction and structural disruption, causing an inward flow of extracellular Ca^2+^, which also leads to calcium overload [73, 74]. Our results further confirmed that BMSCs exhibited mitochondrial alterations and calcium overload under GSDH (Fig. 5F–I) and that these changes were reversed with the addition of AG. Our results confirmed that mitochondrial dysfunction and apoptosis occurred in BMSCs under GSDH and that AG treatment protected against these alterations. Mitochondria are also the powerhouse of the cell and use most of the oxygen to produce ATP to provide energy for cellular life activities. In previous experiments, we found alterations in the mitochondrial morphology and number, and there is recent evidence that AG can regulate glucose metabolism [75]. We further found that after BMSCs were treated with AG under GSDH, cell metabolism changed from glycolysis to oxidative phosphorylation to increase the ATP supply (Fig. 6). This suggests that restoring mitochondrial function in BMSCs in a disease setting may be a cytoprotective mechanism and that modulating mitochondrial quality may help improve the survival of transplanted BMSCs. Taken together, these results provide indirect evidence for the relationship between the antioxidant activity of AG, mitochondrial function and cytoprotective effects. These findings indicate that AG enhanced the survival of BMSCs under GSDH.

The STAT3 and AKT pathways are vital to cell proliferation and resistance to apoptosis [76, 77], and AG has also been reported to be associated with the STAT3 and AKT signaling pathways [75]. However, our results revealed no difference in STAT3 and AKT signaling pathway-related protein expression in BMSCs among the groups (Fig. 7A, B). In our previous study, we found that NRF2 was required for the cellular activity of human-induced pluripotent stem cell-derived cardiomyocytes [46]. It has also been reported that the NRF2 pathway is an important signaling pathway that can resist oxidative stress-induced damage responses, and as a transcription factor, NRF2 can regulate intracellular redox [78, 79]. Activation of NRF2 has been reported to correct the loss of ∆Ψm and is associated with the protection of cells from iron-related death [80]. To determine the further mechanisms of AG on BMSCs survival under GSDH, we revealed that the protein levels of NRF2 and its downstream target proteins NQO1 and HO-1 were significantly increased in the AG group compared to the GSDH group (Fig. 7C, D). Considering that NRF2 exerts its effects by entering the nucleus, we assessed the nuclear level of NRF2 protein, and the results showed that it was significantly increased after AG treatment (Fig. 7E, F). Taken together, we believe that the NRF2 pathways might be responsible for the increased survival of BMSCs under GSDH by AG treatment.

Moreover, BMSCs were treated with ML385, which caused a significant decrease in NRF2 and HO-1 protein levels (Fig. 8A, B). The cell cycle results showed that AG-induced S phase arrest was decreased in the ML385 group; the data investigated that AG promoted the proliferation of BMSCs under GSDH via the NRF2 pathway (Additional file 5: Fig. S5). Analysis of apoptosis and ROS levels further suggested that the protective effect of AG on BMSCs under GSDH could be reversed by ML385 (Fig. 8D, E). The important role of NRF2 in metabolic regulation, in addition to its roles in regulating oxidative stress and cell survival, has received much attention [81], and NRF2 can directly regulate the expression of retinoid X receptor alpha (RXRA) [82], aryl hydrocarbon receptor (AhR) [83, 84], peroxisome proliferator-activated receptor γ (PPARγ) [85, 86] and other metabolically critical genes containing AREs. In addition, metabolism-related enzymes (e.g., pyruvate dehydrogenase kinase isozyme 2, PDK2) are sensitive to ROS, and NRF2 indirectly affects the activity of these metabolic enzymes by regulating ROS, thus changing the flow of energy and affecting metabolism [87]. This may explain the AG-induced alteration in the metabolic pattern of BMSCs under GSDH (Fig. 6) observed in this study, but the exact regulatory mechanism remains to be further confirmed in our future studies, and the detailed mechanisms of the corresponding signaling pathways require further investigation.

In conclusion, we found that AG may effectively inhibit GSDH-induced apoptosis and oxidative stress damage by activating the NRF2 pathway and improve mitochondrial quality to increase the ATP supply for cellular life activities. The important limitation of this study is that in vivo studies are required to determine the efficacy of this approach. However, our data on the protective effect of AG on BMSCs under GSDH will provide new ideas for the transplantation of BMSCs.

## Supplementary Information

**Additional file 1: Fig. S1.** Characterization of BMSCs. (A) Schematic of the BMSCs extraction protocol. (B) Microscopic image showing BMSCs at passage 1 and passage 3. Scale bars: 100 μm.

**Additional file 2: Fig. S2.** The effect of AG on proliferation in BMSCs under GSDH. (A) Chemical structural formula of AG. (B) The CCK-8 assay was performed to measure the viability of BMSCs treated with AG (0, 2, 4, 6, or 8 μM) for 24 h (n = 5). *P  < 0.05, **P  < 0.01, ***P < 0.001, ****P  0. 0001.

**Additional file 3: Fig. S3.** Diagram of the glucose metabolism mechanism.

**Additional file 4: Fig. S4.** Statistical analysis of related proteins in the STAT3 and AKT signaling pathways. Statistical analysis showing the band intensity of p-STAT3/t-STAT3 ratio (A, normalized with β-actin), 14–3-3 ζ/δ/β-actin ratio (B), p-S6/β-actin ratio (C), p-AKT/t-AKT ratio (D, normalized with β-actin), p-FOXO1/t-FOXO1 ratio (E, normalized with β-actin), n = 3. ns, no significance.

**Additional file 5: Fig. S5.** AG regulates the proliferation in BMSCs under GSDH through NRF2 pathway. PI staining was used to measure the cell cycle by flow cytometry and quantitative analysis of the DNA content distribution is shown on the right (n = 3). *P < 0.05.

**Additional file 6: Fig. S6.** Schematic of the potential mechanism by which AG improves the therapeutic effect of BMSCs under GSDH.

## Data Availability

The data in this study are available from the corresponding author upon reasonable request.

## Abbreviations

RT-qPCR: Quantitative reverse transcription polymerase chain reaction
ROS: Reactive oxygen species
AG: Andrographolide
GSDH: Glucose and serum deprivation under hypoxia (1% O_2_)
NRF2: Nuclear factor, erythroid 2-related factor 2
SOD1: Superoxide dismutase 1
GPX4: Glutathione peroxidase 4
HK1: Hexokinase 1
PKM: Pyruvate kinase M1/2
LDH1: Lactate dehydrogenase 1
G6PD: Glucose-6-phosphate dehydrogenase
TKT: Transketolase
TALDO1: Transaldolase 1
CS: Citrate synthase
IDH1: Isocitrate dehydrogenase 1
OGDH: Oxoglutarate dehydrogenase
HO-1: Heme oxygenase 1
NQO-1: NAD(P)H quinone dehydrogenase 1
GSH-px: Glutathione peroxidase 1
CAT: Catalase
GCLC: Glutamate-cysteine ligase catalytic subunit
GAPDH: Glyceraldehyde-3-phosphate dehydrogenase
ND1: NADH dehydrogenase subunit 1
DCFH-DA: 2,7-Dichlorofluorescein diacetate
p-STAT3: Phosphorylated signal transducer and activator of transcription 3
t-STAT3: Total signal transducer and activator of transcription 3
p-AKT: Phosphorylated serine/threonine kinase
t-AKT: Total serine/threonine kinase
p-FOXO1: Phosphorylated forkhead box O1
t-FOXO1: Total forkhead box O1
14-3-3ζ/δ: 14-3-3 Protein zeta/delta
p-S6: Phosphorylated ribosomal protein S6
BMSCs: Bone marrow mesenchymal stem cells
IL-6: Interleukin-6
