# Supplementary figures and images for: Andrographolide protects bone marrow mesenchymal stem cells against glucose and serum deprivation under hypoxia via the NRF2 signaling pathway
Source: Stem Cell Res Ther. 2022 Jul 18;13:326. doi: 10.1186/s13287-022-03016-6 (PMC9290240; doi:10.1186/s13287-022-03016-6)

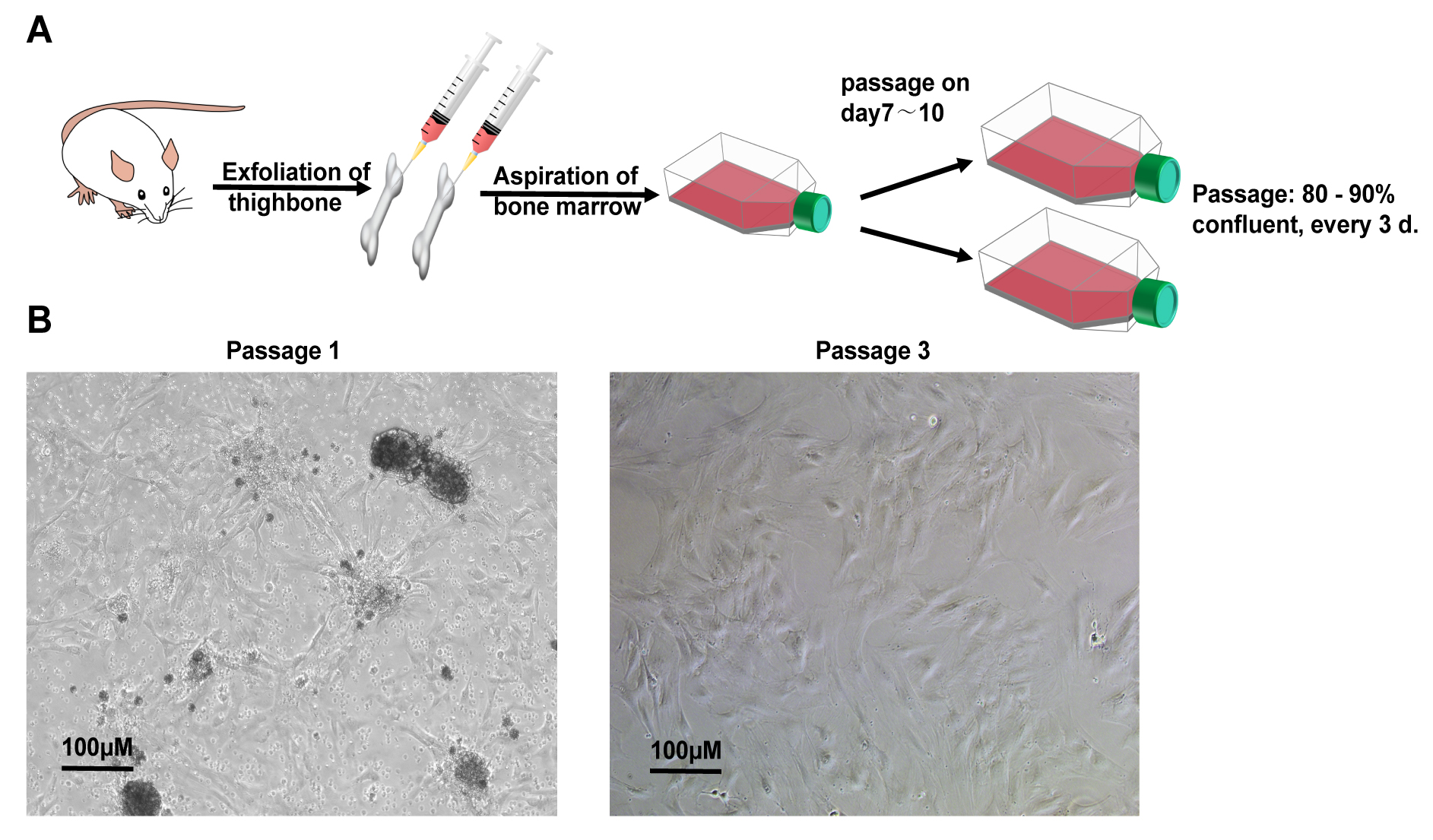

Supplement: Supplementary file 1 — Additional file 1: Fig. S1. Characterization of BMSCs. (A) Schematic of the BMSCs extraction protocol. (B) Microscopic image showing BMSCs at passage 1 and passage 3. Scale bars: 100 μm. [file 13287_2022_3016_MOESM1_ESM.jpg]

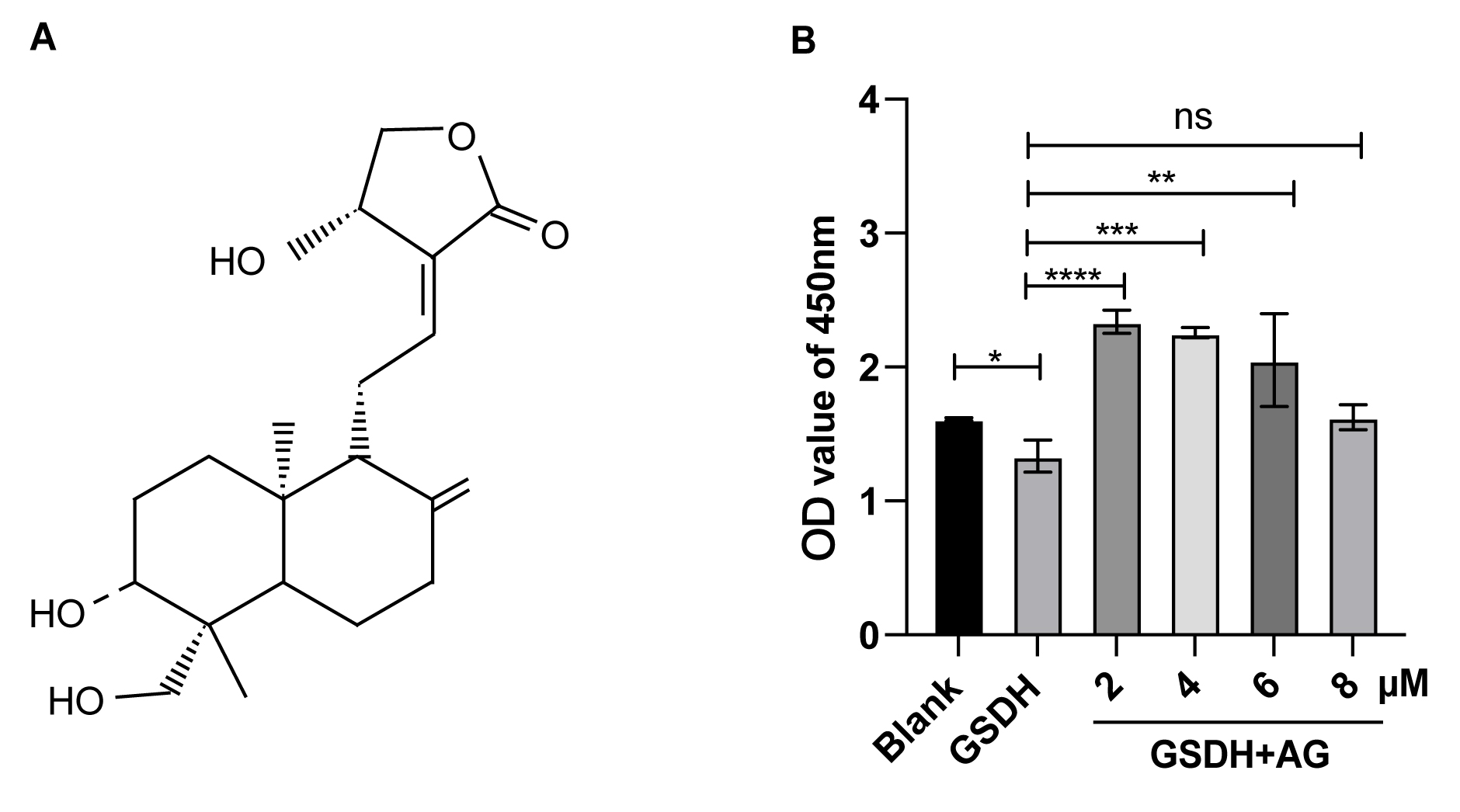

Supplement: Supplementary file 2 — Additional file 2: Fig. S2. The effect of AG on proliferation in BMSCs under GSDH. (A) Chemical structural formula of AG. (B) The CCK-8 assay was performed to measure the viability of BMSCs treated with AG (0, 2, 4, 6, or 8 μM) for 24 h (n = 5). *P < 0.05, **P < 0.01, ***P < 0.001, ****P 0. 0001. [file 13287_2022_3016_MOESM2_ESM.jpg]

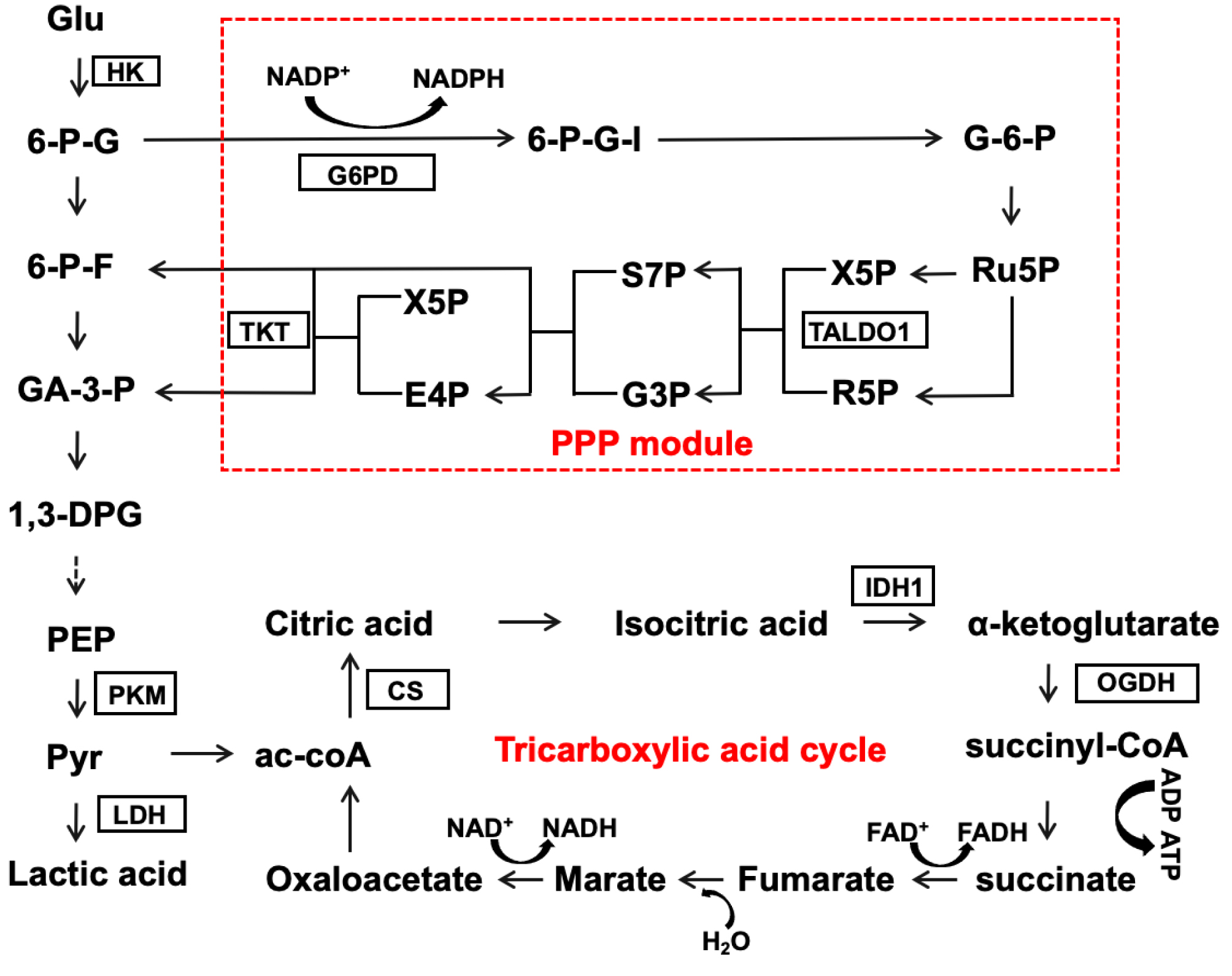

Supplement: Supplementary file 3 — Additional file 3: Fig. S3. Diagram of the glucose metabolism mechanism. [file 13287_2022_3016_MOESM3_ESM.jpg]

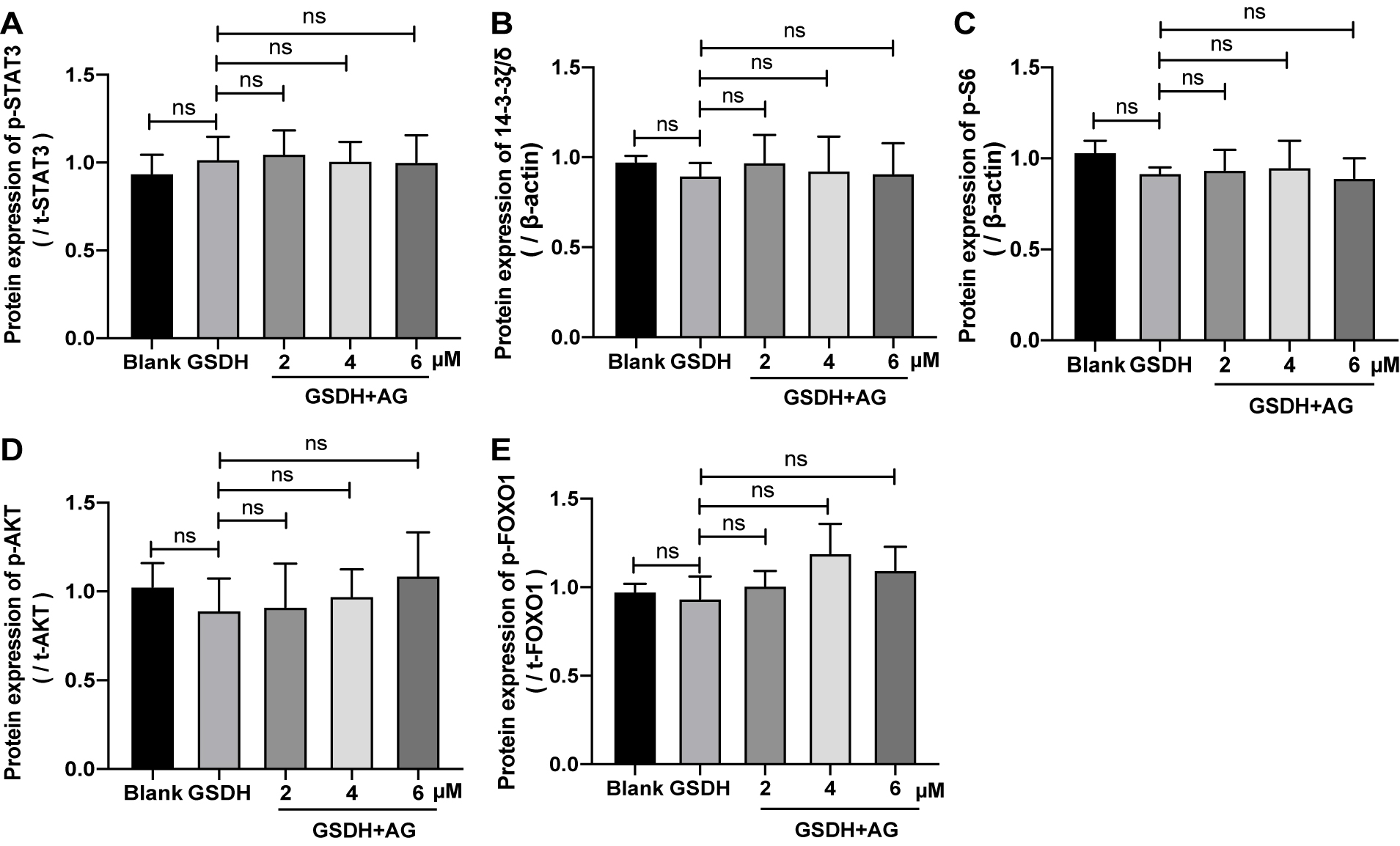

Supplement: Supplementary file 4 — Additional file 4: Fig. S4. Statistical analysis of related proteins in the STAT3 and AKT signaling pathways. Statistical analysis showing the band intensity of p-STAT3/t-STAT3 ratio (A, normalized with β-actin), 14–3-3 ζ/δ/β-actin ratio (B), p-S6/β-actin ratio (C), p-AKT/t-AKT ratio (D, normalized with β-actin), p-FOXO1/t-FOXO1 ratio (E, normalized with β-actin), n = 3. ns, no significance. [file 13287_2022_3016_MOESM4_ESM.jpg]

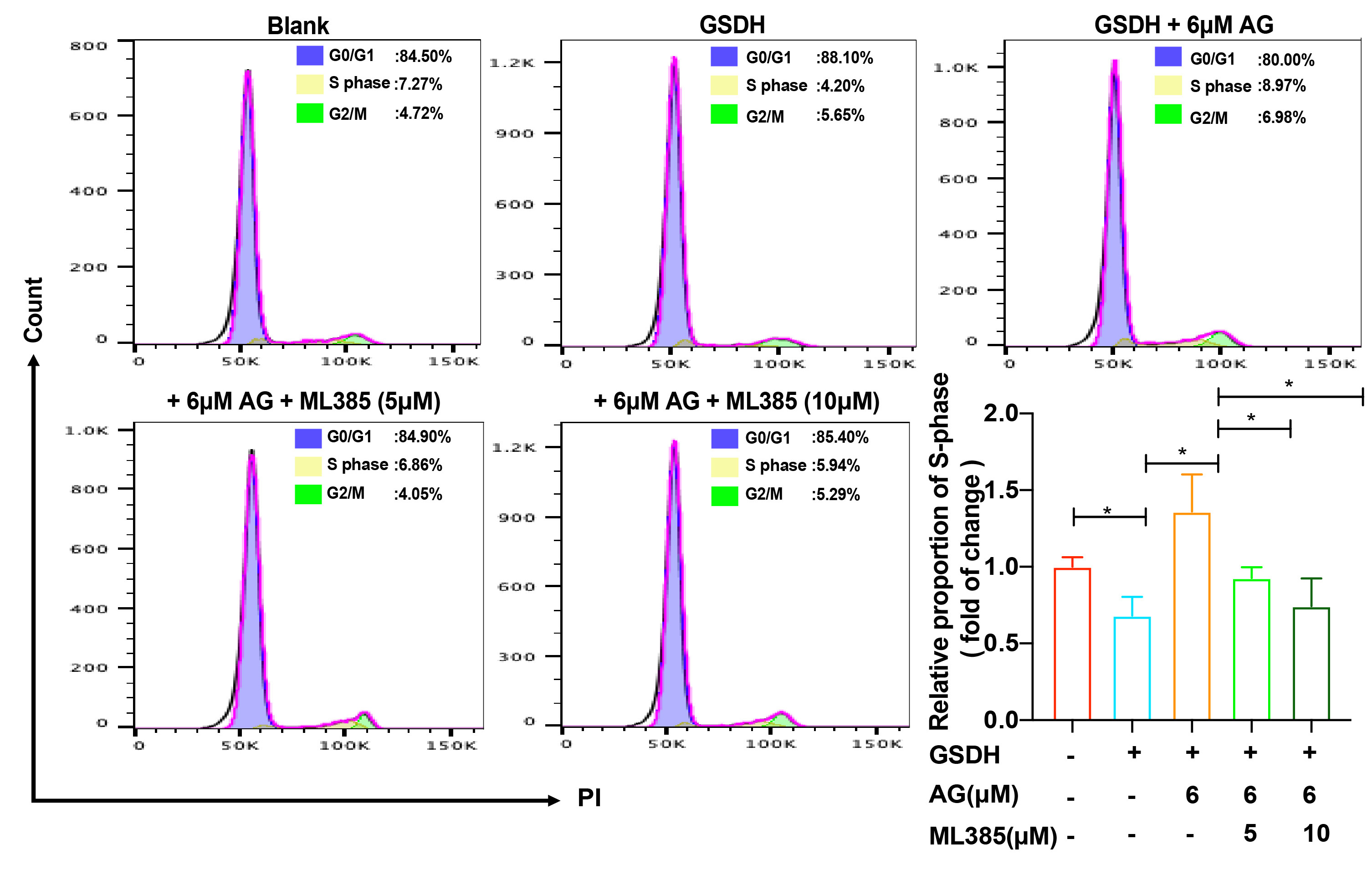

Supplement: Supplementary file 5 — Additional file 5: Fig. S5. AG regulates the proliferation in BMSCs under GSDH through NRF2 pathway. PI staining was used to measure the cell cycle by flow cytometry and quantitative analysis of the DNA content distribution is shown on the right (n = 3). *P < 0.05. [file 13287_2022_3016_MOESM5_ESM.jpg]

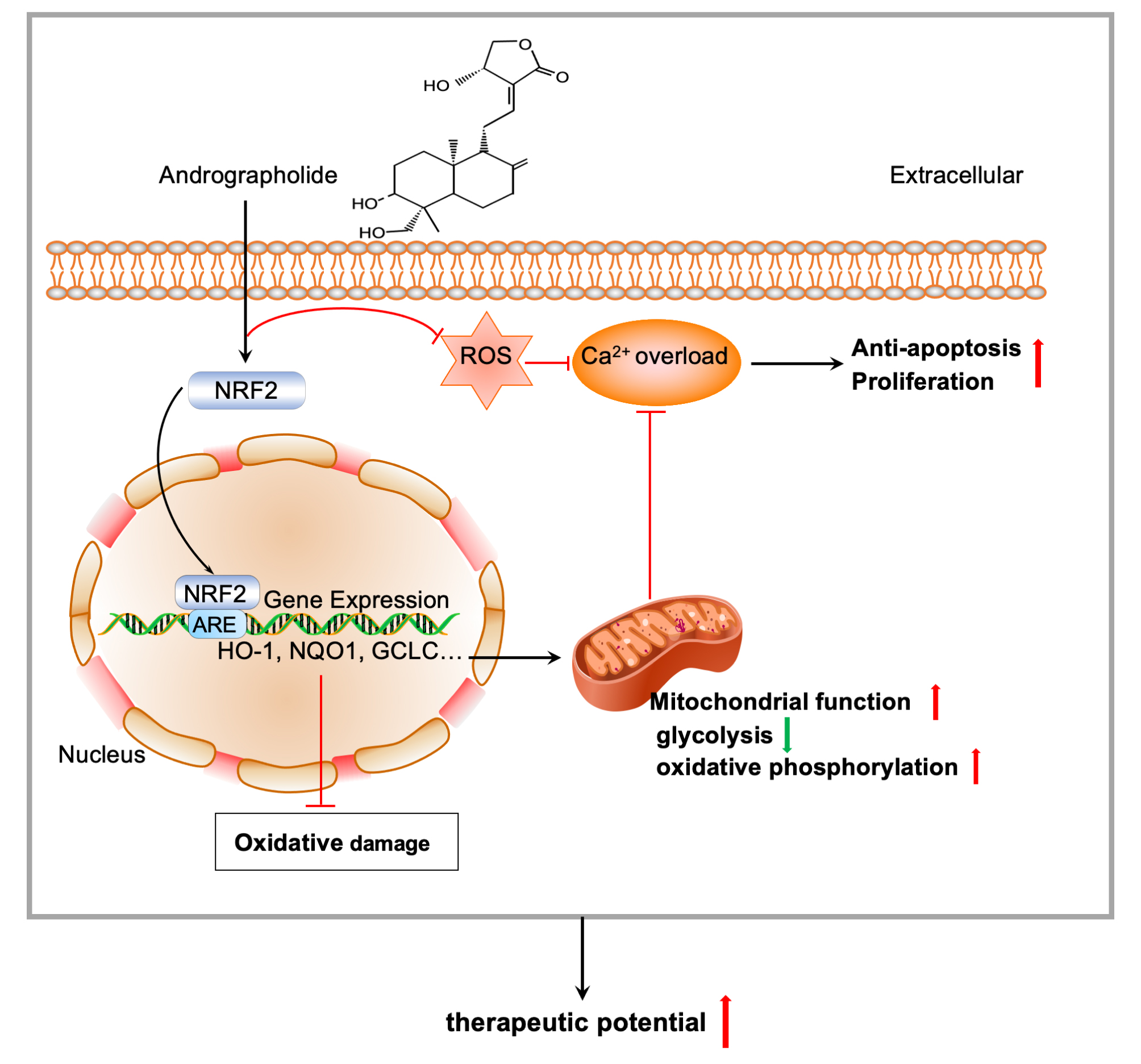

Supplement: Supplementary file 6 — Additional file 6: Fig. S6. Schematic of the potential mechanism by which AG improves the therapeutic effect of BMSCs under GSDH. [file 13287_2022_3016_MOESM6_ESM.jpg]
